# Age-Dependent and Pathway-Specific Bimodal Action of Nicotine on Synaptic Plasticity in the Hippocampus of Mice Lacking the miR-132/212 Genes

**DOI:** 10.3390/cells11020261

**Published:** 2022-01-13

**Authors:** Tamara Stojanovic, David Velarde Gamez, Gabor Jorrid Schuld, Daniel Bormann, Maureen Cabatic, Pavel Uhrin, Gert Lubec, Francisco J. Monje

**Affiliations:** 1 Center for Physiology and Pharmacology, Department of Neurophysiology and Neuropharmacology, Medical University of Vienna, 1090 Vienna, Austria; velardegamez@gmail.com (D.V.G.); n01629958@students.meduniwien.ac.at (G.J.S.); n1118880@students.meduniwien.ac.at (D.B.); maureen.cabatic@meduniwien.ac.at (M.C.); 2 Laboratory for Cardiac and Thoracic Diagnosis, Department of Surgery, Regeneration and Applied Immunology, Medical University of Vienna, Research Laboratories Vienna General Hospital, Waehringer Guertel 18-20, 1090 Vienna, Austria; 3 Division of Thoracic Surgery, Medical University of Vienna, Waehringer Guertel 18-20, 1090 Vienna, Austria; 4 Center for Physiology and Pharmacology, Department of Vascular Biology and Thrombosis Research, Medical University of Vienna, 1090 Vienna, Austria; pavel.uhrin@meduniwien.ac.at; 5 Department of Neuroproteomics, Paracelsus Medical University, 5020 Salzburg, Austria; gert.lubec@lubeclab.com

**Keywords:** miR-132/212, nicotine, ACh, hippocampus, LTP

## Abstract

Nicotine addiction develops predominantly during human adolescence through smoking. Self-administration experiments in rodents verify this biological preponderance to adolescence, suggesting evolutionary-conserved and age-defined mechanisms which influence the susceptibility to nicotine addiction. The hippocampus, a brain region linked to drug-related memory storage, undergoes major morpho-functional restructuring during adolescence and is strongly affected by nicotine stimulation. However, the signaling mechanisms shaping the effects of nicotine in young vs. adult brains remain unclear. MicroRNAs (miRNAs) emerged recently as modulators of brain neuroplasticity, learning and memory, and addiction. Nevertheless, the age-dependent interplay between miRNAs regulation and hippocampal nicotinergic signaling remains poorly explored. We here combined biophysical and pharmacological methods to examine the impact of miRNA-132/212 gene-deletion (miRNA-132/212^−/−^) and nicotine stimulation on synaptic functions in adolescent and mature adult mice at two hippocampal synaptic circuits: the medial perforant pathway (MPP) to dentate yrus (DG) synapses (MPP-DG) and CA3 Schaffer collaterals to CA1 synapses (CA3–CA1). Basal synaptic transmission and short-term (paired-pulse-induced) synaptic plasticity was unaltered in adolescent and adult miRNA-132/212^−/−^ mice hippocampi, compared with wild-type controls. However, nicotine stimulation promoted CA3–CA1 synaptic potentiation in mature adult (not adolescent) wild-type and suppressed MPP-DG synaptic potentiation in miRNA-132/212^−/−^ mice. Altered levels of CREB, Phospho-CREB, and acetylcholinesterase (AChE) expression were further detected in adult miRNA-132/212^−/−^ mice hippocampi. These observations propose miRNAs as age-sensitive bimodal regulators of hippocampal nicotinergic signaling and, given the relevance of the hippocampus for drug-related memory storage, encourage further research on the influence of miRNAs 132 and 212 in nicotine addiction in the young and the adult brain.

## 1. Introduction

Nicotine addiction, as characterized by sustained and uncontrolled tobacco smoking, is behind millions of deaths worldwide and is causally related to conditions, such as coronary heart failure, atherosclerosis, brain stroke, and also to malignancies (such as oesophagus, lung, colon, and liver cancers) [1,2,3,4,5,6]. Psychological interventions and pharmacotherapeutic attempts to handle nicotine addiction are poor in success, reflecting our scarcity of knowledge about the neurocognitive and molecular/functional effects of nicotine on the synaptic circuits underlying drug addiction. Given the associated human suffering and social cost, understanding the molecular and functional underpinnings of the action of nicotine in the brain neural circuitry is of paramount biomedical and scientific importance.

The initiation of nicotine intake behaviors in humans (smoking in particular), as well as the onset of certain smoking-related psychiatric disorders, mostly begins during adolescence [7,8,9,10,11,12,13], thus highlighting the importance of understanding the relevance of age as a factor critically influencing the impact of nicotine on brain functions. Similar to during childhood, the maturation of brain circuits regulating emotions and higher order cognitive functions (including learning and memory) undergo major developmental changes and marked adaptations during adolescence in response to external stimuli [14,15,16,17,18,19,20,21]. In particular, the hippocampus present with pronounced morpho-functional transformations during adolescence and is importantly involved in nicotine addiction and learning and memory [15,20,22,23,24,25].

MicroRNAs (miRNAs) are short-length noncoding RNA transcripts that exert modulatory roles in gene expression by acting on protein-coding messenger RNAs (mRNAs), either to block their translation or to induce their destabilization and degradation [26,27]. Rising evidence proposes miRNAs as regulators of hippocampal synaptic plasticity [28,29,30,31,32,33] and as modulators of neuroadaptations in the context of addiction [34,35]. The miRNAs 132 and 212, which share a genetically codifying intron in a gene found at the chromosome number 17 in humans, have been previously shown to be associated to the activity of both BDNF and CREB, i.e., two molecular elements that play central roles in synaptic plasticity and memory functions [36,37,38,39,40,41], thus suggesting a role for these two miRNAs in synaptic plasticity and learning and memory functions. Indeed, several groups, including ours, have observed that the deletion of genes encoding for the miRNAs 132/212 results in altered synaptic plasticity and mood, as well as memory functions [30,42,43,44,45,46,47,48].

Acetylcholine receptors are distributed across the mammalian brain and are abundantly expressed in brain regions relevant for memory storage and nicotine addiction [49,50,51,52,53]. Previous reports have showed a functional crosstalk between members of the miRNA 132/212 complex with elements of the brain acethylcolinergic signaling pathway [54,55,56]. Indeed, our group had previously described that stimulation with nicotine results in abolishment of hippocampal synaptic plasticity in mice lacking the genes encoding for the miRNAs 132 and 212 plasticity [44]. The involvement of the miRNA-132/212 cluster in synaptic transmission and plasticity has also been previously associated to glutamatergic synaptic transmission in LTP experiments (see for example [44,46,57]); to learning and memory and BDNF, CREB, and MeCP2 signaling in vivo (see for example [36]); to altered levels of Abeta peptide, which is associated to Alzheimer’s disease [58]; and to other brain neuronal synaptic functions, as described by our group and our close collaborators [30,42,45]. However, despite the fact that miRNAs and acethylcolinergic signaling regulate synaptic plasticity and mediate in the addictive response, the mechanisms by which addictive drugs influence the structural and functional remodeling of the hippocampal neuronal circuitry in an age-dependent manner remains poorly explored. We here examined the effects of nicotine stimulation on two different hippocampal synaptic circuits and at two different ages using, as an experimental animal model, the miRNA-132/212 knockout mice (miRNA-132/212^−/−^) derived from the line generated by the group of Dr. Yuriy Pankratov, who moreover had presented pioneering results which describe the involvement of these microRNA family in hippocampal synaptic plasticity [44]. We here provide experimental evidence proposing that miR-132/212 participate, in an age-dependent manner, in the regulation of the hippocampal responses to nicotine at selective neuronal circuits known to be relevant for both nicotine addiction and memory storage.

## 2. Materials and Methods

### 2.1. Animals

We here used male C57Bl/6 wild-type (WT) and previously described [44] miRNA-132/212 knockout (miRNA-132/212^−/−^) mice (adolescent cohort was 5–9 weeks old, mature adult cohort was 17–25 weeks old). WT littermates served as controls and genotypes verified by PCR. Housing conditions and food and water administration were comparable to that of our previous works [30,42]. This study observed ethical BMWF-66.009/0200-WF/V/3b/2016 Austrian guidelines. The wellbeing of all the animals was monitored by expert veterinarians. ARRIVE and U.K. Animals Usage Guidelines (Scientific Procedures Act, 1986 and associated guidelines, as well as EU Directive 2010/63/EU for animal experiments) were heeded.

### 2.2. Immunoblotting

Methods for brain tissue isolation of miRNA-132/212^−/−^ and WT mice (here, 5 animals per group were used), as well as the process of immunoblotting, have been previously described by our group [30,42]. Antibodies: M1-mAChR (1:1000; Cat. Nr.: M9808 Sigma-Aldrich Handels GmbH, Vienna, Austria); AChE (1:1000; Cat. Nr.: ab183591 Abcam); CREB (1:1000; Cat. Nr.: 9197 Cell Signalling Technology, Danvers, MA, USA); pCREB (1:1000; Cat. Nr.: 9198 Cell Signalling Technology, Danvers, MA, USA); goat-anti rabbit (1:3000; Cat. Nr.: 7074S Cell Signalling Technology); GAPDH (1:3000; Cat Nr. MA5-15738 Thermo Fisher Scientific, Vienna, Austria).

### 2.3. Electrophysiology

#### 2.3.1. Hippocampal Slices Preparation

Slices from hippocampi (400 µm thick) were prepared as previously described by our group [30,42]. Here, 5–6 animals were used per experimental group.

#### 2.3.2. Extracellular Recordings

**Figure 1:**
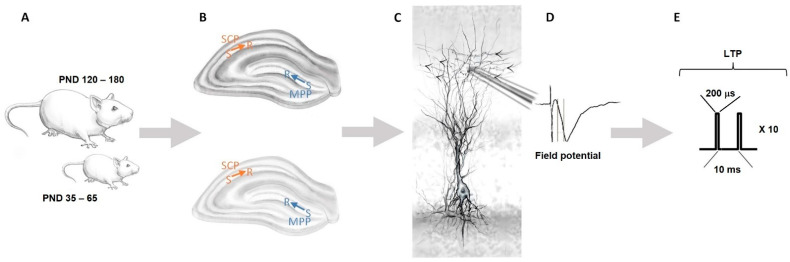
Diagram of the experimental setting for slice electrophysiology. (**A**) Age-specific effects of CholinomiRs interplay in CNS were addressed by comparing responses of adolescent mice (PND 35–65) and adult mice (PND 120–180). (**B**) Illustrations of hippocampal slices from old and young mice. The place where stimulating (S) and recording (R) electrodes were located at the MPP (blue) and SCP (orange) are indicated. Simulation was delivered at the middle inputs coming from the entorhinal cortex molecular layer for recordings at the dentate gyrus and at the CA3 stratum radiatum layer for recordings at the SCP. (**C**) Graphic detail of an electrode used for recordings positioned at a dendritic zone. (**D**) Illustrative field potential recording. (**E**) Drawing for the pattern of pulses of the electrical stimulation used to induce LTP (see also Section 2). PND: postnatal day; MPP: medial perforant pathway; SCP: Schaffer collateral pathway; LTP: long-term potentiation.

Slice electrophysiology and recordings at the hippocampal stratum radiatum were conducted as in our previous reports and those from colleagues [30,42,59,60]. fEPSPs recordings from the dentate gyri and from the CA1 regions were conducted as described before by our group [30,42,61,62]. Analysis of basal synaptic transmission (input/output (I/O) curves) and plasticity was conducted as described before [30,42] (see also Figure 1). To induce LTP, we applied 20 pulses of electrical stimulation (200 μs/pulse) at 100 Hz (Figure 1). Paired-pulse-induced synaptic plasticity (facilitation (PPF) or inhibition (PPI)) was recorded and analyzed as described in [30]. Stimulation protocols were delivered at baseline stimulation intensities. LTP was determined by analyzing changes in the decaying phase of fEPSP slopes after high frequency stimulation, normalized to baseline. Data were averaged within groups and compared between miRNA-132/212^−/−^ and WT mice, with or without nicotine presence in the recording chamber. In some experiments, slices were simulated twice for 5 min (with a 5 min interval) with bath-applied aCSF solution containing 1 µM nicotine (N5260, Sigma Aldrich) as used in independent experimental settings described before [63,64,65]. In particular, we here decided to use a double nicotine-stimulation protocol, as we previously described that this stimulation protocol can acutely induce changes in hippocampal plasticity [30]. Previous reports have shown effects on plasticity upon brief exposures to nicotine preceding the application of stimulation protocols inducing LTP [66], and other groups have also described the use of brief and intermittent stimulations with molecules that activate neuronal cell-surface receptors in in vitro settings in order to induce morphological and functional neuronal responses in studies of synaptic plasticity and memory-related functions [67]. Electrical stimulation was generated from an ISO-STIM 01D (NPI Electronics, Tamm, Germany). An AxoClamp-2B amplifier, a Digidata-1440 interface (Axon Instruments, Molecular Devices, Berkshire, UK), and pClamp-11 software (Molecular Devices, Berkshire, UK) were used for the acquisition and analysis of data.

### 2.4. Statistical Analysis

GraphPad Prism-7.0 (San Diego, CA, USA) was used for analysis. Sample data distribution was weighed before statistical analyses by D’Agostino’s K^2^ normality test. The differences between datasets from two groups were evaluated with a two-sided unpaired Student’s *t*-test. To conduct multiple comparisons, we used repeated-measures (or mixed model) ANOVA with Bonferroni’s test. Data in the figures show values of means with standard errors (+/−). A value of 0.05 was used as level of significance (α).

## 3. Results

### 3.1. Basal Synaptic Transmission and Paired-Pulse-Induced Plasticity Is Unaltered in Adolescent and Mature Adult miRNA-132/212^−/−^ Mouse Hippocampi

Artificially induced alterations in the frequency of activity at specific hippocampal presynaptic terminals (using electrical stimulation) can result in “plastic” changes characterized by either strengthening (synaptic potentiation; inducible by high-frequency stimulation) or dwindling (synaptic depression; inducible by low-frequency stimulation) the postsynaptic response. The magnitude and persistence in time of the elicited change in the postsynaptic response directly relates to the frequency and intensity of the delivered electrical stimulation [68,69,70,71]. These experimental observations prompted neuroscientists to propose that perhaps the plastic changes associated to memory storage in the mammalian brain could operate following functional principles analogue to those elicited artificially [72,73,74,75,76,77,78]. However, while several modalities of neuronal plasticity are described in ex vivo studies that used brain slices and standardized protocols of electrical stimulation (see for example [30,79]), a lot is yet to be understood about the molecular elements concomitantly modulating hippocampal synaptic plasticity, memory storage, and nicotine addiction.

We, therefore, explored the effects of brain maturation and miRNA-132/212 gene deletion on paired-pulse-induced hippocampal synaptic plasticity at MPP-DG (blue in Figure 1B) and CA3–CA1 (orange in Figure 1B) synapses. Field potential recordings were obtained from slices derived from male adolescent and adult miRNA-132/212 gene knockout (miRNA-132/212^−/−^), as well as on their relative littermate wild-type counterparts used as controls (Figure 1). The effect of 1 μM nicotine was examined.

**Figure 2:**
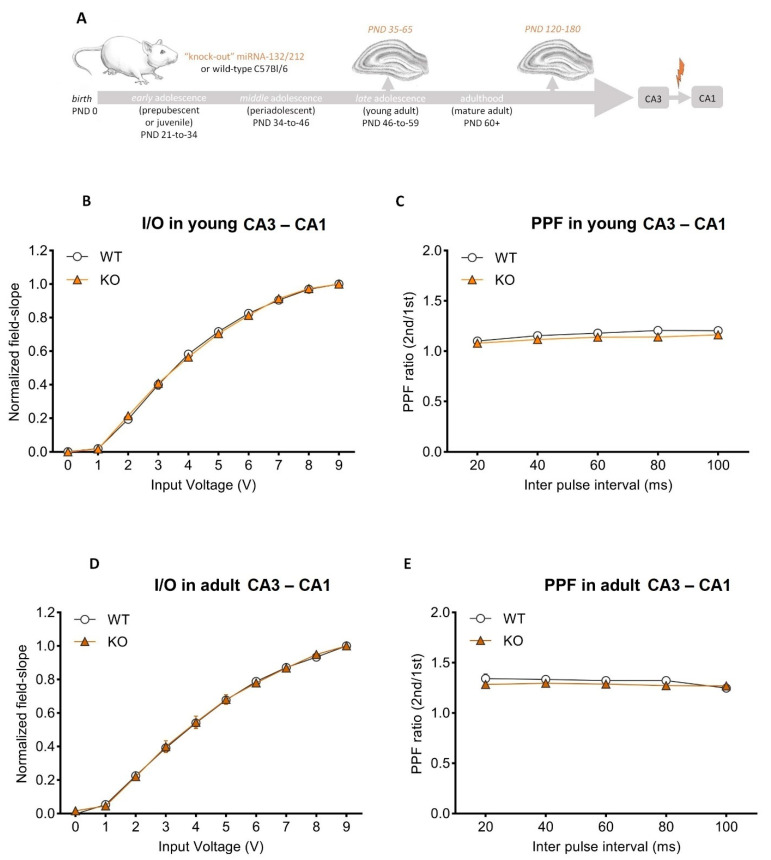
Unaltered basal synaptic transmission and paired-pulse facilitation in adolescent or adult hippocampal CA3–CA1 synapses of miRNA-132/212 knockout mice. (**A**) Schematic study design and experimental approach to study LTP induction at the Schaffer collateral output synapses. (**B**) Averaged field slopes vs. stimulation voltages (as derived from I/O protocols) for adolescent (PND 35–65) wild-type (WT; *n* = 16) and miRNA-132/212^−/−^ mice (KO, *n* = 12). Data showed no genotype differences (ns = *p* < 0.05; ANOVA (repeated measures) was followed by the Bonferroni test; error bars presented as SEM). (**C**) Averaged PPF ratios vs. increasing inter-pulse intervals. No differences are apparent between groups (ns = *p* < 0.05; ANOVA (repeated measures) followed by the Bonferroni test; error bars presented as SEM). (**D**) Averaged field slopes vs. increasing stimulation voltages (from I/O protocols) in mature-adult (PND 120–180) wild-type (WT, *n* = 11) and miRNA-132/212^−/−^ (KO, *n* = 9) slices. No statistical differences between genotypes were apparent (ns = *p* < 0.05; ANOVA (repeated measures) followed by the Bonferroni test; error bars presented as SEM). (**E**) Averaged PPF ratios vs. increasing inter-pulse intervals. No differences were detected between the groups (ns = *p* < 0.05; ANOVA (repeated measures) was followed by the Bonferroni test; error bars presented as SEM). PPF: Paired-Pulse Facilitation; PND: postnatal day. For I/O, ANOVA for adolescent cohort indicated an effect of stimulation voltage (*p* < 0.0001, F (1.232, 32.03) = 2301) without genotype effect (*p* = 0.9885, F (1, 26) = 0.0002112), and for adult cohort a predicted main significant effect of input voltage (*p* > 0.0001, F (1.451, 26.12) = 1286) but no significant effect of genotype (*p* = 0.9042, F (1, 18) = 0.01490). For paired-pulse-induced plasticity; (ANOVA for adolescent cohort revealed main effect of inter-pulse interval (*p* < 0.0001, F (2.507, 65.19) = 39.82) without genotype effects (*p* = 0.2207, F (1, 26) = 1.575); without effects of the time interval between pulses (*p* = 0.2253, F (1.275, 22.94) = 1.585) and no effects of the genotype (*p* = 0.2561, F (1, 18) = 1.376) for the for adult cohort.

**Figure 3:**
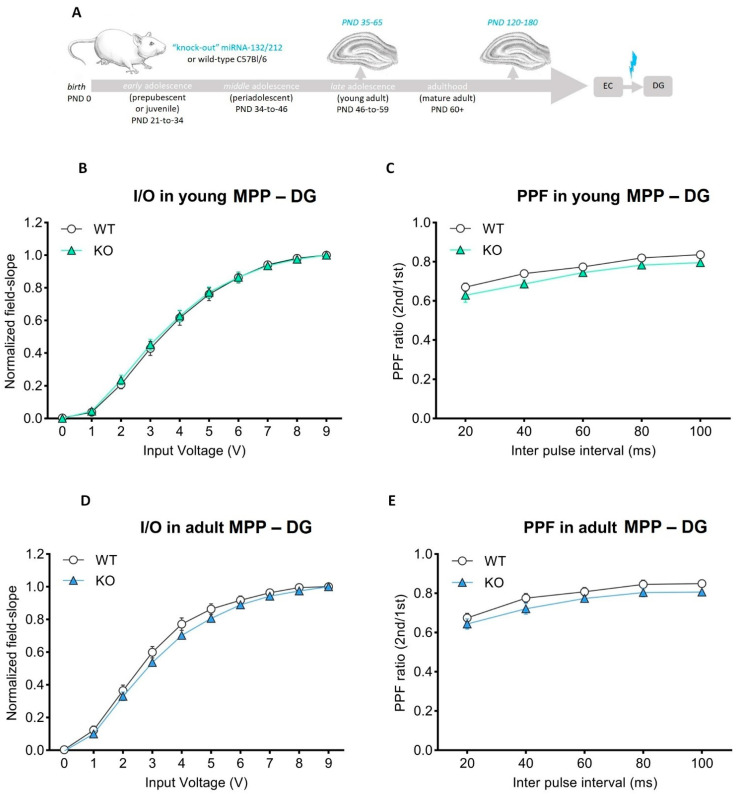
Basal synaptic transmission and paired-pulse-induced inhibition are unaltered in the adolescent or adult dentate gyrus of miRNA-132/212 knockout mice. (**A**) Schematic study design and experimental approach to study LTP induction at the dentate gyrus output synapses. (**B**) Averaged field slope vs. voltage stimulation (resulting from I/O protocols) in adolescent (PND 35–65) wild-type (WT, *n* = 10) and in miRNA-132/212^−/−^ (KO, *n* = 10) slices. No differences were apparent between groups (ns = *p* < 0.05; ANOVA (repeated measures) was followed by the Bonferroni test; error bars presented as SEM). (**C**) Averaged PPI ratios vs. increasing inter-pulse intervals. No differences were detected between the groups (ns = *p* < 0.05; ANOVA (repeated measures) was followed by the Bonferroni test; error bars presented as SEM). (**D**) Averaged field slope values vs. stimulation voltage (from I/O protocols) for adult (PND 120–180) wild-type (WT, *n* = 11) and miRNA-132/212^−/−^ (KO, *n* = 16) slices showed no differences between genotype (ns = *p* < 0.05; ANOVA (repeated measures) was followed by the Bonferroni test; error bars presented as SEM). (**E**) Averaged PPI ratios vs. increasing inter-pulse intervals. No differences were apparent between the groups (ns = *p* < 0.05; ANOVA (repeated measures) was followed by the Bonferroni test; error bars presented as SEM). PPF: paired-pulse facilitation; MPP-DG: medial perforant path—dentate gyrus; PND: postnatal day. For I/O and paired-pulse induced plasticity; two-way repeated measures ANOVA for adolescent young adults I/O data revealed main effects of stimulation voltage (*p* < 0.0001, F (1.176, 21.18) = 926.8) without genotype effect (*p* = 0.8096, F (1, 18) = 0.05977) and for the mature adult cohort a predicted main inter-pulse interval effect (*p* < 0.0001, F (1.406, 35.15) = 1211) without significant effect of genotype (*p* = 0.1435, F (1, 25) = 2.281). Similarly, ANOVA for paired-pulse-induced plasticity experiments in adolescent young adults also showed significant effect of the time-interval between pulses (*p* < 0.0001, F (1.869, 33.64) = 83.13) without genotype effect (*p* = 0.1370, F (1, 18) = 2.423). For the mature adult cohort, also a main significant effect of inter-pulse interval (*p* < 0.0001, F (2.640, 66.01) = 129.1) with no significant effect of genotype was observed (*p* = 0.2079, F (1, 25) = 1.671).

Before hand, we used input/output protocols (Section 2) to assess basal synaptic transmission at CA3–CA1 synapses (Figure 2A). Recordings from either miRNA-132/212^−/−^ or WT groups yielded I/O responses comparable for miRNA-132/212^−/−^ and either adolescent or adult WT groups (Figure 2B,D). These results provide experimental evidence indicating that miRNA-132/212^−/−^ are not required for the proper expression of basal synaptic transmission and voltage stimulation sensitivity, i.e., an observation somewhat different from previous reports [44] and likely attributable to the differences in the age of the used animals and other experimental conditions (e.g., differences can be attributable to types of recording chambers, pattern of stimulation protocol, recovery conditions, etc. [80,81,82]). We then studied paired-pulse-induced synaptic plasticity for both experimental groups and conditions. Slice recordings from nicotine-free (untreated) miRNA-132/212^−/−^ showed paired-pulse-induced field slopes, in CA3–CA1 synapses comparable to those of WT controls, regardless of their age (Figure 2C,E). These observations ruled out a requirement of miRNA-132/212 for the impact that nicotine has on proper short-term potentiation in CA3–CA1 synapses in adolescent and adult male mice. We subsequently examined the properties of both synaptic transmission (basal activity) and paired-pulse-induced plasticity at MPP-DG hippocampal synapses of adolescent and mature adult mice (miRNA-132/212^−/−^ vs. WTs (Figure 3A)). Recordings for both groups were statistically indistinguishable, irrespective of their age (Figure 3B–E).

### 3.2. Nicotine Bolsters Synaptic Potentiation at CA3–CA1 Synapses of Mature Adult, Not Adolescent Wild-Type Mice

**Figure 4:**
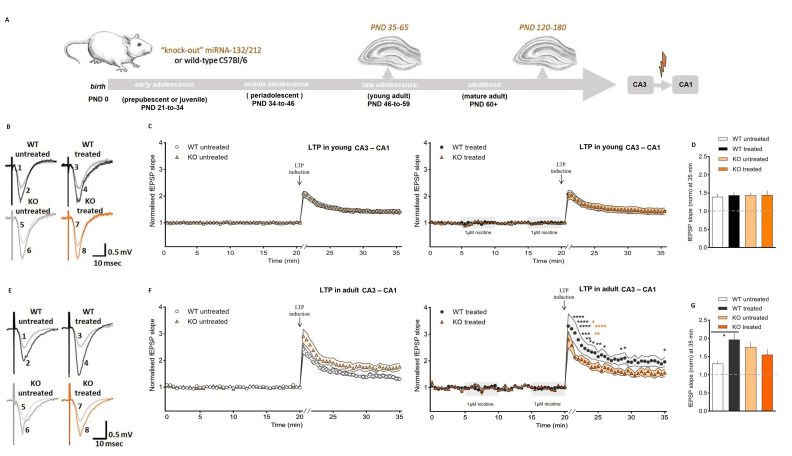
Nicotine enhances LTP at CA3–CA1 synapses of adult but not adolescent wild-type mice. (**A**) Schematic study design and experimental approach to study LTP induction at the Schaffer collateral output synapses. (**B**) Demonstrative fEPSPs during base line recordings (indicated with numbers 1, 3, 5, and 7) and after inducing LTP (indicated with numbers 2, 4, 6, and 8), as obtained in slices from young adolescent (PND 35–65) wild-type and miRNA-132/212^−/−^ animals (in both cases, with and without nicotine treatment). LTP traces are from the 21 min (initial response to potentiation induction). (**C**) fEPSPs slope changes before and after inducing LTP in slices derived from young adolescent untreated wild-type (WT untreated, *n* = 8) and miRNA-132/212^−/−^ (KO untreated *n* = 6) mice (left panel). On the right panel, data obtained after nicotine treatment in slices from wild-type (WT treated, *n* = 7) and miRNA-132/212^−/−^ (KO treated, *n* = 6) animals are also shown. The application of 1 μM nicotine before the induction of LTP (two applications of 5 min with a 5 min interval of washout) is represented by shadowed boxes in the right panel. A salient, comparable increase in synaptic potentiation was apparent in the recordings from both untreated and nicotine-treated wild-type and miRNA-132/212^−/−^ groups (ns = *p* < 0.05; ANOVA (repeated measures) was followed by the Bonferroni test; error bars presented as SEM). (**D**) Normalized fEPSP amplitudes at 35 min (ns = *p* > 0.05; ANOVA (repeated measures) was followed by the Bonferroni test; error bars presented as SEM). ANOVA analysis (Mixed-effects) revealed statistical significance in the effect of elapsed time (*p* < 0.0001, F (70, 910) = 119.4) without significant impact from the genotype (*p* = 0.8619, F (1, 13) = 0.03148) and the nicotine treatment (*p* = 0.7977, F (1, 13) = 0.06845). ANOVA with the Bonferroni post hoc analysis of the values at min 35, after inducing LTP, did not show group differences (untreated WT versus treated WT: ns *p* > 0.05, t 23 = 0.3644; untreated WT versus untreated miRNA-132/212^−/−^ (KO): ns *p* > 0.05, t 23 = 0.3632; treated WT versus treated miRNA-132/212^−/−^ (KO): ns *p* > 0.05, t 23 = 0.04914; untreated miRNA-132/212^−/−^ (KO) versus miRNA-132/212^−/−^ (KO) treated: ns *p* > 0.05, t 23 = 0.03419). (**E**) Demonstrative fEPSPs, from base line (numbers 1, 3, 5 and 7) and after LTP induction (numbers 2, 4, 6, and 8), as recorded in slices derived from mature adult (PND 120–180) untreated wild-type and miRNA-132/212^−/−^ mice (both groups with and without nicotine treatment). LTP traces are from the 21 min (initial response to potentiation induction). (**F**) fEPSPs slope changes before and after inducing LTP in slices derived from mature adult (PND 120–180) untreated wild-type (WT untreated, *n* = 5) and miRNA-132/212^−/−^ (KO untreated, *n* = 3) mice (left panel) and for slices from wild-type slices treated with nicotine (WT treated, *n* = 6) as well as for slices from miRNA-132/212^−/−^ mice, also treated with nicotine (KO treated, *n* = 6) in the right panel. Nicotine was applied as for the young animals described above (and as indicated by the shadowed boxes in the figure). Robust but comparable LTP responses were observed in slices from miRNA-132/212^−/−^ mice, untreated or treated with nicotine. Untreated slices from wild-type mice also showed robust LTP. However, a significant enhancement of LTP was detected in response to nicotine only in slices from wild-type mice (black asterixis represent significant differences in synaptic potentiation between nicotine-treated WT and untreated WT groups, while orange asterixis (set to the right-hand side) between nicotine-treated WT and nicotine-treated miRNA-132/212^−/−^ groups; ANOVA (repeated measures) was followed by the Bonferroni test; error bars show SEM). Augmentation in LTP upon nicotine treatment of adult wild-type mice and no effect of nicotine treatment on LTP of adolescent wild-type mice (**C**,**F**) is observable. (**G**) Normalized fEPSP amplitudes at 35 min (*p* < 0.05; ANOVA (repeated measures) was followed by the Bonferroni test; error bars are representing SEM). LTP: long-term potentiation; PND: postnatal day. ANOVA (Mixed-effects) revealed statistical significance in the effect of time (*p* < 0.0001, F (70, 630) = 64.41) without genotype effects (*p* = 0.5986, F (1, 9) = 0.2976) and nicotine treatment (*p* = 0.2949, F (1, 9) = 1.237; (**F**)). ANOVA with post hoc Bonferroni analysis of values at 35 min revealed significant group differences (WT vs. WT treated: *p* = 0.0289, t 16 = 3.270) without statistically significant effects observed between the other experimental conditions examined (WT versus miRNA-132/212^−/−^: *p* > 0.05, t 16 = 1.872; WT nicotine-treated versus miRNA-132/212^−/−^ nicotine-treated: *p* > 0.05, t 16 = 0.8667; miRNA-132/212^−/−^ versus miRNA-132/212^−/−^ nicotine-treated: *p* > 0.05, t 16 = 0.8953; (**G**)).

Long-term potentiation (LTP), as experimentally defined by the plastic ability of synapses to undergo stimuli-induced functional strengthening, has been proposed as a potential mechanism for memory formation in vivo [76,83,84,85,86,87,88,89,90]. In both young and adult mammalian brains, LTP can be experimentally elicited in regions, such as the hippocampal MPP-DG and the CA3–CA1 area, and many authors have also established a link between LTP and addiction [91,92,93,94,95,96,97]. In order to examine the potential age-dependent participation of miRNA-132/212 in the modulation of LTP and the response to nicotine exposure, we obtained hippocampal slices from adolescent and mature adult mice (WT vs. miRNA-132/212^−/−^) and examined the nicotine impact on LTP in developing and fully mature synapses (Figure 4A). All adolescent mice slices, WT and miRNA-132/212^−/−^, showed comparable synaptic potentiation responses. Next, we exposed the adolescent slices from both groups to the double exposure (5-min each time) of 1-μM nicotine. The bath-applied double stimulation with nicotine had no detectable effects on the LTP responses, regardless of genotype (Figure 4B–D).

We next examined field potential responses in hippocampal slices from mature adult mice (WT and miRNA-132/212^−/−^). Slices obtained from both groups of untreated mature adult mice showed fEPSP responses similar to those observed in their adolescent counterparts, with a rather enhanced LTP response observed only for the adult miRNA-132/212^−/−^ relative to the other groups, suggesting a tendency to enhanced LTP, which is possibly attributable to aging-related phenomena still remaining to be clarified. Additionally, no significant effects of the treatment with nicotine were apparent in slices from adult miRNA-132/212^−/−^ mice, which also showed a rather enhanced LTP relative to the responses observed in their young counterparts but not larger than that observed in the adult untreated miRNA-132/212^−/−^ group, thus indicating that adult miRNA-132/212^−/−^ have a promoted LTP response in the CA1 region, somewhat analogue to previous observed experiments [44] but without changes in the sensitivity to nicotine stimulation. However, a robust and significant enhancement of synaptic potentiation was observed in mature adult WT mice slices that were treated with nicotine (Figure 4E–G). Note the circa 25% enhancement in LTP upon nicotine treatment of mature adult WT mice (Figure 4E–G) versus the lack of effect on LTP upon treatment with nicotine in adolescent WT mice (Figure 4B–D). Interestingly, in slices obtained from mature adult WT mice, the effects of nicotine on CA3–CA1 synapses became prominent immediately following the induction of LTP (that is, during post-tetanic potentiation (PTP)), with fEPSPs remaining enhanced for the entire time of electrophysiological assessment (Figure 4F). On the contrary, slices obtained from adolescent WT mice remained unresponsive to the effect of the treatment with the paired 5-min exposure to 1 μM of nicotine throughout the entire electrophysiological assessment (Figure 4C).

### 3.3. Nicotine Shifts from Promoter to Suppressor of Synaptic Potentiation in the Dentate Gyrus in Absence of miRNA-132/212

The dentate gyrus importantly influences learning and memory, comprises a brain niche for neurogenesis, and is markedly affected by nicotine, as this alkaloid alters the process of synaptic plasticity [23,98,99]. Recently, our group unveiled the existence of a functional interplay between miRNAs and nicotinergic signaling that appears to be involved in the regulation of the expression of cell-surface membrane receptors and that participates in the modulation of neuroplasticity in the mature, fully developed hippocampus [30]. However, the possible role of the miRNAs 132 and 212 as age-dependent regulators of the hippocampal responses to nicotine remain, to the best of our knowledge, unexplored.

**Figure 5:**
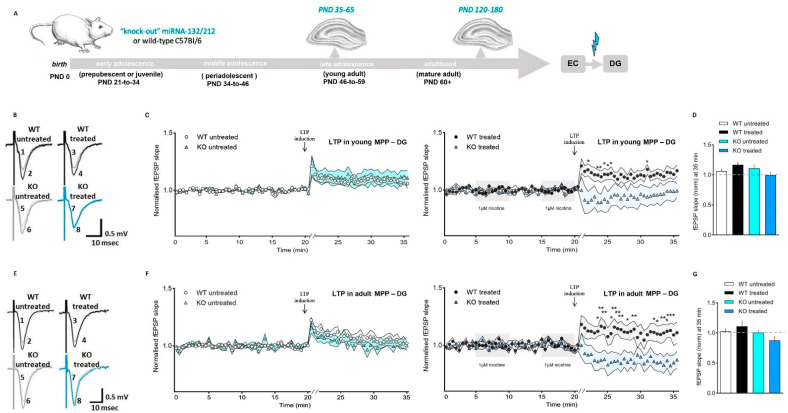
Nicotine abolishes LTP in the dentate gyrus of mice lacking the miRNA-132/212 genes. (**A**) Schematic study design and experimental approach to study LTP induction at the dentate gyrus output synapses. (**B**) Demonstrative fEPSPs from base line measurements (numbers 1, 3, 5, and 7) and after LTP induction (numbers 2, 4, 6, and 8), in slices from adolescent (PND 35–65) control wild-type (WT untreated) and miRNA-132/212^−/−^ (KO untreated) animals, and corresponding nicotine-treated wild-type (WT treated) and nicotine-treated miRNA-132/212^−/−^ (KO treated). fEPSPs traces for LTP are from 21 min (initial response to potentiation). (**C**) fEPSPs slope change before and after LTP induction in slices from adolescent (PND 35–65) untreated WT (*n* = 5) and untreated miRNA-132/212^−/−^ (KO; *n* = 5) mice (left panel) and in slices from corresponding nicotine-treated WT (*n* = 5) and nicotine-treated miRNA-132/212^−/−^ (KO; *n* = 5) mice (right panel). Nicotine treatment (1 μM) was delivered as for the previous experiments (see shadowed boxes in the right panel). A robust, significant LTP enhancement was detectable in slices from both WT and untreated miRNA-132/212^−/−^ animals, whereas LTP was virtually eliminated in slices from nicotine-treated miRNA-132/212^−/−^ mice (ANOVA (repeated measures) was followed by the Bonferroni test; error bars represent SEM). Untreated groups are presented in the left panel and nicotine-treated are presented in the right panel. (**D**) Normalized fEPSP amplitudes at min 35, after inducing LTP, (ns = *p* < 0.05; ANOVA (repeated measures) was followed by the Bonferroni test; error bars show SEM). Mixed-effects ANOVA yielded a significant time effect (*p* < 0.0001, F (70, 560) = 6.867) without the genotype effect (ns *p* = 0.2425, F (1, 8) = 1.593) and treatment (ns *p* = 0.2118, F (1, 8) = 1.841). ANOVA analysis with the Bonferroni post hoc test of the values at min 35, after inducing LTP, found no group differences (untreated WT versus nicotine-treated WT: ns *p* > 0.05, t 16 = 1.403; untreated WT versus untreated miRNA-132/212^−/−^: ns *p* > 0.05, t 16 = 0.6243; nicotine-treated WT versus nicotine-treated miRNA-132/212^−/−^: ns *p* > 0.05, t 16 = 2.244; untreated miRNA-132/212^−/−^ versus nicotine-treated miRNA-132/212^−/−^: ns *p* > 0.05, t 16 = 1.466). (**E**) Demonstrative fEPSP traces from base line (numbers 1, 3, 5 and 7) and after LTP induction (numbers 2, 4, 6, and 8), from mature adult (PND 120–180) WT (treated and untreated) and miRNA-132/212^−/−^ (KO; treated and untreated) hippocampal slices. Responses for LTP correspond to 21 min. (**F**) fEPSP slope changes before and after LTP induction in slices from adult (PND 120–180) WT (untreated, *n* = 5) and miRNA-132/212^−/−^ mice (KO; untreated, *n* = 9) (left panel) and corresponding WT (nicotine-treated, *n* = 6) and miRNA-132/212^−/−^ mice (KO; nicotine-treated, *n* = 7) in the right panel. Nicotine was applied as described in figures above (shadowed boxes in the figure right panel). Augmented LTP was apparent in recordings from untreated WT and untreated KO mice, whereas LTP was obliterated in nicotine-treated KO mice slices (black asterisks indicate LTP differences between nicotine-treated WT and nicotine-treated KO groups, whereas blue asterisks indicate LTP differences between nicotine-treated KO and untreated KO groups; ANOVA (repeated measures) was followed by the Bonferroni test; error bars show SEM). (**G**) Normalized fEPSP amplitudes at min 35, after inducing LTP, (ns = *p* > 0.05; ANOVA (repeated measures) was followed by the Bonferroni test. ANOVA analysis revealed a time effect (*p* < 0.0001, F (70,980) = 1.865) as well as a significant effect for genotype and for treatment (*p* < 0.0001, F (70,625) = 3.601). ANOVA analysis with the Bonferroni post hoc test of the values at min 35, after LTP induction, showed no group differences (untreated WT versus nicotine-treated WT: ns *p* > 0.05, t 23 = 0.8301; untreated WT versus untreated miRNA-132/212^−/−^: ns *p* > 0.05, t 23 = 0.2079; nicotine-treated WT versus nicotine-treated miRNA-132/212^−/−^: ns *p* > 0.05, t 23 = 2.471; untreated miRNA-132/212^−/−^ versus nicotine-treated miRNA-132/212^−/−^: ns *p* > 0.05, t 23 = 1.500; (**G**)). Error bars are shown as SEM). MPP-DG: medial perforant path—dentate gyrus; PND: postnatal day.

We, hence, studied the impact of nicotine exposure on synaptic potentiation in young developing and fully mature MPP-DG synapses using slices from adolescent and mature adult WT and miRNA-132/212^−/−^ mice (Figure 5A). To this aim, we used conventional electrophysiological methods previously proven to efficiently induce short- and long-term forms of synaptic potentiation in slices from mice and rat hippocampi [81,100,101,102]. Field-slope responses in untreated slices from adolescent mice from both groups (WT and miRNA-132/212^−/−^) showed comparable forms of synaptic strengthening in MPP-DG synapses.

In contrast, whereas slices from adolescent WT animals treated with 1 μM of nicotine (two bath applications given with 5-min intervals) showed markedly promoted synaptic strengthening, the slices from adolescent miRNA-132/212^−/−^ animals showed no potentiation at all and exhibited features resembling those observed after application of pulses of electrical stimulation inducing synaptic depression (Figure 5B–D).

We subsequently examined the field potential responses in MPP-DG synapses in slices from mature-adult mice, WT, and miRNA-132/212^−/−^. Under untreated control conditions, both groups showed synaptic potentiation responses without significant differences (Figure 5E–G) and comparable to the responses under untreated conditions of adolescent mice as displayed in Figure 5B–D. However, in slices obtained from adult mice the response to 1 μM of nicotine resulted in virtually abolished memory-related synaptic strengthening in miRNA-132/212^−/−^ mice (Figure 5E–G), an effect paralleling that observed in adolescent miRNA-132/212^−/−^ mice, as shown in Figure 5B–D. Nicotine treatment of mature adult WT mice, on the other hand, resulted in slight yet enhanced synaptic potentiation (Figure 5F), an effect observed in adolescent WT mice (compare black circle symbol in Figure 5C,F).

### 3.4. Altered Expression Levels of CREB, pCREB and AChE in the Adult Hippocampus of miRNA-132/212 Knockout Mice

**Figure 6:**
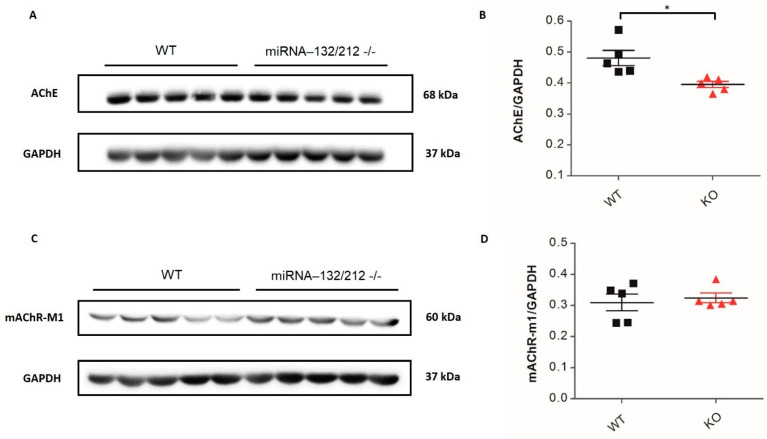
The hippocampal protein levels of acetylcholinesterase but not M1-mAChR are altered in miRNA-132/212^−/−^ mice. Western blot from hippocampal tissue was used to assess the levels of expression of the proteins AChE and mAChR-M1. (**A**) Demonstrative blots and (**B**) relative expression of AChE and GAPDH, showing a significant decrease in AChE in miRNA-132/212^−/−^ mice, in comparison to WT mice (* = *p* < 0.05). (**C**) Illustrative blots and (**D**) relative protein expression of mAChR-M1 and GAPDH, showing no differences for mAChR-M1 in miRNA-132/212^−/−^ tissue compared to WT (*p* > 0.05; *t*-test). Results are shown as fold change normalized to GAPDH protein levels. Data are shown as mean ± SD, *n* = 5/group. kDa = kilodalton.

**Figure 7:**
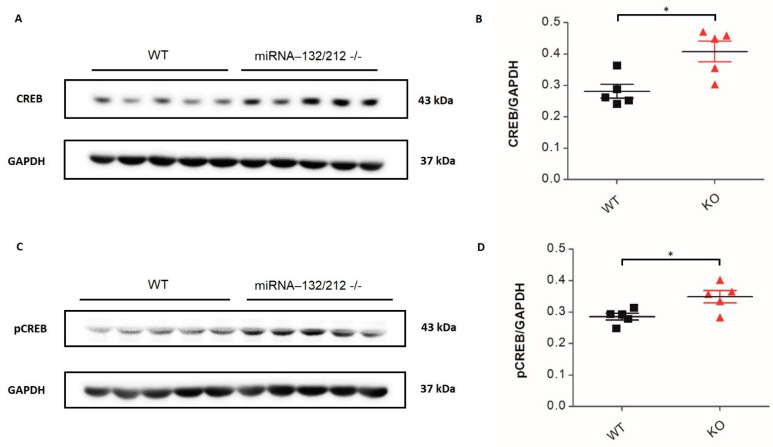
Knockdown of the miRNAs 132 and 212 genes alters protein levels and phosphorylation of CREB in adult mouse hippocampus. Western blots from hippocampal tissues were used to examine the levels of total and phosphorylated CREB. (**A**) Demonstrative blots and (**B**) expression levels of total CREB and GAPDH, showing significant enhancement of total CREB in miRNA-132/212^−/−^ (KO), in comparison to wild type (WT) (* = *p* < 0.05; *t*-test). (**C**) Illustrative blots and (**D**) expression levels of phosphorylated CREB and GAPDH showing significant enhancement of phosphorylated CREB in miRNA-132/212^−/−^ tissue in comparison to WT (* = *p* < 0.05; *t*-test). Results are shown as fold change normalized to GAPDH protein levels. Data are shown as mean ± SD, *n* = 5/group. kDa = kilodalton.

We next examined the levels of expression of the proteins acetylcholinesterase (AChE), i.e., M1 muscarinic acetylcholine receptor (M1-mAChR), cAMP response element-binding protein (CREB), and phosphorylated CREB (pCREB) in the hippocampus of miRNA-132/212^−/−^ mice by conventional Western blot densitometric analyses, as previously described [30,42]. Data showed a significant reduction (*p* = 0.0126; t 8 = 3.201; *t*-test) in AChE for the miRNA-132/212^−/−^ group relative to the WT control group (Figure 6A,B), whereas M1-mAChR showed no differences (ns *p* = 0.6465; t 8 = 0.4765; *t*-test) between groups (Figure 6C,D). Additionally, both total CREB (Figure 7A,B) and pCREB (Figure 7C,D) were enhanced in miRNA-132/212^−/−^ hippocampi relative to WT controls (*p* = 0.0130; t 8 = 3.179; *t*-test and *p* = 0.0225; t 8 = 2.820; *t*-test, respectively).

## 4. Discussion

The hippocampus of humans and rodents share a large variety of genetic, structural, functional, and pharmacological commonalities and also overlap in key events in maturation during adolescence and adulthood [14,15,20]. While the time scale of the central nervous system developmental phases is considerably different between human and murine species, rodents, however, also exhibit a remarkable vulnerability to nicotine addiction at to human corresponding age stages (e.g., adolescence) and also share numerous features when addiction-related physiological and behavioral changes are examined [14,15,103,104,105,106,107,108,109,110]. The signaling elements that become altered and consolidated in the young brain upon nicotine exposure remain, however, poorly understood. Combining molecular biological, pharmacological, and biophysical studies in murine animal models can, therefore, importantly aid in the search for the identification of those still missing molecular elements influencing the brain neuronal vulnerability to nicotine at specific stages of brain development. Using this experimental approach, we here provide experimental evidence proposing an age-dependent and pathway-specific role for miRNA-132/212 in the modulation of the action of nicotine on memory-related hippocampal synaptic functions. Our data further reveal altered acetylcholinesterase and CREB signaling in miRNA132/212 knockout mice, thus encouraging further independent research on these initial, potential molecular elements that could participate in vivo, via miRNA-132/212 regulation, in the brain neuronal response to nicotine.

## 5. Acetylcholinergic Signaling and Hippocampal Synaptic Plasticity

We here have studied the effects of nicotine on hippocampal synaptic plasticity in the young and adult wild-type and miRNA-132/212^−/−^ mice and further examined the expression levels of pCREB, AChE, and M1 in the hippocampi of miRNA-132/212^−/−^ mice. It must be noted, however, that nicotine is, of course, not degraded by AChE and does not target M1 receptors. We examined the levels of M1 receptors in order to expand our knowledge on the association between miRNAs 132/212 and members of the acethylcolinergic signaling complex since we had previously reported that miRNA-132/212^−/−^ mice presented with augmented protein levels of the α7-nAChR [30]. The reported unaltered levels of M1 receptors in untreated miRNA-132/212^−/−^ mice hippocampi, therefore, suggest a potential specificity for the involvement of these microRNAs in the selective regulation of acethylcolinergic pathways. It would be also interesting to explore the effect of nicotine on the levels of AChE in the hippocampus of miR-132/212 knockout mice in future experiments, as nicotine has been proposed as a possible inhibitor of brain AChE [111].

Abundant scientific literature has previously established the impact of nicotinic cholinergic activity on LTP in a variety of hippocampal pathways, including CA3–CA1 synaptic circuits [112,113,114,115,116,117,118] and MPP-DG synapses [30,119,120,121,122]. At the CA3–CA1 synaptic circuit, nicotine was also shown to promote LTP in the aged brain [114]. In hippocampal slices, 1 μM of nicotine facilitated LTP induction, while having no effect on baseline fEPSPs [117,123]. On these grounds, we here also applied 1 μM of nicotine and studied its effects on hippocampal LTP in MPP-DG as well as on CA3–CA1 synapses. Our experimental data, derived from the use of a previously characterized knockout mice model [44], for the first time propose a potential role for the microRNAs 132 and 212 in the modulation of the effects that nicotine has on hippocampal synaptic plasticity in both pathway-specific and age-dependent manners. Our observations, thus, suggest the miRNA-132/212 complex as potential in vivo modulator of the selective effects on nicotine on addiction- and memory-related behaviors in the adolescence versus the adulthood through region-specific effects modulated across the life span.

The participation of nicotinic acetylcholine receptor [25,114,124,125,126,127,128,129] and of the acetylcholinesterase [130,131] in hippocampal synaptic functions has been long established (see also [132,133,134]) [135]. Several groups had also described the relevance of CREB in hippocampal synaptic plasticity and memory-related functions [40,76,136,137] and in nicotinergic signaling [24,138,139,140]. The miRNA-132/212 family had also been implicated in regulation of synaptic functions [30,44,45,46], dendritic growth and arborization of hippocampal neurons [141], and higher-order executive functions that are necessary for the cognitive control of behavior [36,47,142]. Previous studies had also shown that miR132 is a target of CREB and that the expression of miRNA-132 is linked to CREB and ERK1/2 activity [37,143]. However, neither the functional crosstalk between the actions of nicotine and of acetylcholinesterase and CREB signaling through miRNA-132/212, or the relevance of age and neural circuitry for these effects, had been previously established. Our group had previously shown that Erk levels are altered in mice lacking the genes encoding for the miRNAs 132 and 212 [30]. Since Erk and CREB are concomitantly associated with both hippocampal synaptic plasticity and memory functions [144,145,146,147,148], we therefore hypothesize that miRNA132/212 might mediate in the modulation of those learning- and memory-related functions that become activated in vivo upon sustained early nicotine exposure through nicotinic acetylcholine receptors, acetylcholinesterase, and Erk/CREB signaling. The relevance of both age and neuronal pathway, apart from the differences introduced through the specific experimental protocols used, can further lie beneath the bimodal actions mediated by miRNA-132/212 and on the diverse alterations in the synaptic function upon deletion of the miRNA-132/212 genes [30,44,149,150]. Given the relevance of the hippocampus in memory functions, miRNA-132/212 advantageous position at the molecular level, thus, emerged through our work as a potential temporal–spatial regulator of nicotine-related gene expression at specific brain developmental stages to influence hippocampal neural plasticity and associated behavioral changes during drug-related memory formation and maintenance.

## 6. Limitations

It must be noted that, in this work, only male animals were used, and there are important differences reported for several neuronal properties, including synaptic plasticity and memory functions, that are strongly dependent on the sex of the animals used in this type of studies [61,151,152,153,154]. Therefore, our data here should not be generalized to the female population. For example, some physiological processes and related diseases (e.g., Parkinson’s disease) can be differentially modulated in a sex-dependent manner [155,156,157,158]. Similar considerations should be taken in account to avoid a generalization about the scope of our data on the grounds of the possible influence of factors, such as the strain [159,160,161,162]. We, thus, encourage additional independent research to expand our observations on female subjects and with different strains, models, and ages.

## 7. Conclusions

Experimental data provided here suggest the existence of an in vivo, fine-tuned age- and pathway-specific mechanism of modulation of the effects of the endogenous neurotransmitter acetylcholine on hippocampal synaptic plasticity. This work expands our current scope of neuronal signaling mechanisms potentially implicated in drug addiction and proposes miR-132/212 as a potential target for pharmacotherapeutic studies searching for ways to ameliorate the deleterious effects of nicotine on the remodeling of neuronal circuits relevant for learning acquisition, memory retention, and drug addiction consolidation. Further research and independent experimental verification of our data might, thus, contribute to enhance our knowledge on the molecular and functional mechanisms determining the age-dependent and pathway specific functional crosstalk between microRNAs and nicotine addiction.

## Data Availability

All the data generated and/or analyzed in this study has been included in this article.

## References

[1] Benowitz N.L. (2010). Nicotine addiction. N. Engl. J. Med..

[2] Lien G., DeLand K. (2011). Translating the WHO Framework Convention on Tobacco Control (FCTC): Can we use tobacco control as a model for other non-communicable disease control?. Public Health.

[3] Yach D. (2014). The origins, development, effects, and future of the WHO Framework Convention on Tobacco Control: A personal perspective. Lancet.

[4] Jha P. (2015). Deaths and taxes: Stronger global tobacco control by 2025. Lancet.

[5] Ezzati M., Lopez A.D. (2003). Estimates of global mortality attributable to smoking in 2000. Lancet.

[6] Makate M., Whetton S., Tait R.J., Dey T., Scollo M., Banks E., Norman R., Pidd K., Roche A.M., Allsop S. (2020). Tobacco Cost of Illness Studies: A Systematic Review. Nicotine Tob. Res..

[7] Kandel D., Yamaguchi K. (1993). From beer to crack: Developmental patterns of drug involvement. Am. J. Public Health.

[8] Kandel D.B., Yamaguchi K. (1985). Developmental patterns of the use of legal, illegal, and medically prescribed psychotropic drugs from adolescence to young adulthood. NIDA Res. Monogr..

[9] Resnick M.D., Bearman P.S., Blum R.W., Bauman K.E., Harris K.M., Jones J., Tabor J., Beuhring T., Sieving R.E., Shew M. (1997). Protecting adolescents from harm. Findings from the National Longitudinal Study on Adolescent Health. JAMA.

[10] Hanna E.Z., Yi H.Y., Dufour M.C., Whitmore C.C. (2001). The relationship of early-onset regular smoking to alcohol use, depression, illicit drug use, and other risky behaviors during early adolescence: Results from the youth supplement to the third national health and nutrition examination survey. J. Subst. Abus..

[11] Lai S., Lai H., Page J.B., McCoy C.B. (2000). The association between cigarette smoking and drug abuse in the United States. J. Addict. Dis..

[12] Barron S., White A., Swartzwelder H.S., Bell R.L., Rodd Z.A., Slawecki C.J., Ehlers C.L., Levin E.D., Rezvani A.H., Spear L.P. (2005). Adolescent vulnerabilities to chronic alcohol or nicotine exposure: Findings from rodent models. Alcohol. Clin. Exp. Res..

[13] Biederman J., Monuteaux M.C., Mick E., Wilens T.E., Fontanella J.A., Poetzl K.M., Kirk T., Masse J., Faraone S.V. (2006). Is cigarette smoking a gateway to alcohol and illicit drug use disorders? A study of youths with and without attention deficit hyperactivity disorder. Biol. Psychiatry.

[14] Spear L. (2000). Modeling adolescent development and alcohol use in animals. Alcohol. Health.

[15] Spear L.P. (2000). The adolescent brain and age-related behavioral manifestations. Neurosci. Biobehav. Rev..

[16] Giedd J.N. (2004). Structural magnetic resonance imaging of the adolescent brain. Ann. N. Y. Acad. Sci..

[17] Giedd J.N., Blumenthal J., Jeffries N.O., Castellanos F.X., Liu H., Zijdenbos A., Paus T., Evans A.C., Rapoport J.L. (1999). Brain development during childhood and adolescence: A longitudinal MRI study. Nat. Neurosci..

[18] Steinberg L. (2005). Cognitive and affective development in adolescence. Trends Cogn. Sci..

[19] Suzuki M., Hagino H., Nohara S., Zhou S.Y., Kawasaki Y., Takahashi T., Matsui M., Seto H., Ono T., Kurachi M. (2005). Male-specific volume expansion of the human hippocampus during adolescence. Cereb. Cortex.

[20] Arain M., Haque M., Johal L., Mathur P., Nel W., Rais A., Sandhu R., Sharma S. (2013). Maturation of the adolescent brain. Neuropsychiatr. Dis. Treat..

[21] Wierenga L., Langen M., Ambrosino S., van Dijk S., Oranje B., Durston S. (2014). Typical development of basal ganglia, hippocampus, amygdala and cerebellum from age 7 to 24. NeuroImage.

[22] Gould T.J. (2006). Nicotine and hippocampus-dependent learning: Implications for addiction. Mol. Neurobiol..

[23] Cohen A., Soleiman M.T., Talia R., Koob G.F., George O., Mandyam C.D. (2015). Extended access nicotine self-administration with periodic deprivation increases immature neurons in the hippocampus. Psychopharmacology.

[24] Fisher M.L., LeMalefant R.M., Zhou L., Huang G., Turner J.R. (2017). Distinct Roles of CREB Within the Ventral and Dorsal Hippocampus in Mediating Nicotine Withdrawal Phenotypes. Neuropsychopharmacology.

[25] Kenney J.W., Gould T.J. (2008). Modulation of hippocampus-dependent learning and synaptic plasticity by nicotine. Mol. Neurobiol..

[26] Ambros V. (2001). microRNAs: Tiny regulators with great potential. Cell.

[27] Bartel D.P. (2004). MicroRNAs: Genomics, biogenesis, mechanism, and function. Cell.

[28] Edbauer D., Neilson J.R., Foster K.A., Wang C.F., Seeburg D.P., Batterton M.N., Tada T., Dolan B.M., Sharp P.A., Sheng M. (2010). Regulation of synaptic structure and function by FMRP-associated microRNAs miR-125b and miR-132. Neuron.

[29] Oliver R.J., Mandyam C.D. (2018). Regulation of Adult Neurogenesis by Non-coding RNAs: Implications for Substance Use Disorders. Front. Neurosci..

[30] Stojanovic T., Benes H., Awad A., Bormann D., Monje F.J. (2020). Nicotine abolishes memory-related synaptic strengthening and promotes synaptic depression in the neurogenic dentate gyrus of miR-132/212 knockout mice. Addict. Biol..

[31] Baby N., Alagappan N., Dheen S.T., Sajikumar S. (2020). MicroRNA-134-5p inhibition rescues long-term plasticity and synaptic tagging/capture in an Abeta(1-42)-induced model of Alzheimer’s disease. Aging Cell.

[32] Berentsen B., Patil S., Ronnestad K., Goff K.M., Pajak M., Simpson T.I., Wibrand K., Bramham C.R. (2020). MicroRNA-34a Acutely Regulates Synaptic Efficacy in the Adult Dentate Gyrus In Vivo. Mol. Neurobiol..

[33] Zhang H.P., Liu X.L., Chen J.J., Cheng K., Bai S.J., Zheng P., Zhou C.J., Wang W., Wang H.Y., Zhong L.M. (2020). Circulating microRNA 134 sheds light on the diagnosis of major depressive disorder. Transl. Psychiatry.

[34] Chandrasekar V., Dreyer J.L. (2009). microRNAs miR-124, let-7d and miR-181a regulate cocaine-induced plasticity. Mol. Cell Neurosci..

[35] Chen C.L., Liu H., Guan X. (2013). Changes in microRNA expression profile in hippocampus during the acquisition and extinction of cocaine-induced conditioned place preference in rats. J. Biomed. Sci..

[36] Hernandez-Rapp J., Smith P.Y., Filali M., Goupil C., Planel E., Magill S.T., Goodman R.H., Hebert S.S. (2015). Memory formation and retention are affected in adult miR-132/212 knockout mice. Behav. Brain Res..

[37] Remenyi J., Hunter C.J., Cole C., Ando H., Impey S., Monk C.E., Martin K.J., Barton G.J., Hutvagner G., Arthur J.S. (2010). Regulation of the miR-212/132 locus by MSK1 and CREB in response to neurotrophins. Biochem. J..

[38] Nudelman A.S., DiRocco D.P., Lambert T.J., Garelick M.G., Le J., Nathanson N.M., Storm D.R. (2010). Neuronal activity rapidly induces transcription of the CREB-regulated microRNA-132, in vivo. Hippocampus.

[39] Numakawa T., Richards M., Adachi N., Kishi S., Kunugi H., Hashido K. (2011). MicroRNA function and neurotrophin BDNF. Neurochem. Int..

[40] Barco A., Patterson S., Alarcon J.M., Gromova P., Mata-Roig M., Morozov A., Kandel E.R. (2005). Gene expression profiling of facilitated L-LTP in VP16-CREB mice reveals that BDNF is critical for the maintenance of LTP and its synaptic capture. Neuron.

[41] Patterson S.L., Pittenger C., Morozov A., Martin K.C., Scanlin H., Drake C., Kandel E.R. (2001). Some forms of cAMP-mediated long-lasting potentiation are associated with release of BDNF and nuclear translocation of phospho-MAP kinase. Neuron.

[42] Bormann D., Stojanovic T., Cicvaric A., Schuld G.J., Cabatic M., Ankersmit H.J., Monje F.J. (2021). miRNA-132/212 Gene-Deletion Aggravates the Effect of Oxygen-Glucose Deprivation on Synaptic Functions in the Female Mouse Hippocampus. Cells.

[43] Pichler S., Gu W., Hartl D., Gasparoni G., Leidinger P., Keller A., Meese E., Mayhaus M., Hampel H., Riemenschneider M. (2017). The miRNome of Alzheimer’s disease: Consistent downregulation of the miR-132/212 cluster. Neurobiol. Aging.

[44] Remenyi J., van den Bosch M.W., Palygin O., Mistry R.B., McKenzie C., Macdonald A., Hutvagner G., Arthur J.S., Frenguelli B.G., Pankratov Y. (2013). miR-132/212 knockout mice reveal roles for these miRNAs in regulating cortical synaptic transmission and plasticity. PLoS ONE.

[45] Ronovsky M., Zambon A., Cicvaric A., Boehm V., Hoesel B., Moser B.A., Yang J., Schmid J.A., Haubensak W.E., Monje F.J. (2019). A role for miR-132 in learned safety. Sci. Rep..

[46] Aten S., Hansen K.F., Hoyt K.R., Obrietan K. (2016). The miR-132/212 locus: A complex regulator of neuronal plasticity, gene expression and cognition. RNA Dis..

[47] Hansen K.F., Karelina K., Sakamoto K., Wayman G.A., Impey S., Obrietan K. (2013). miRNA-132: A dynamic regulator of cognitive capacity. Brain Struct. Funct..

[48] Hansen K.F., Sakamoto K., Aten S., Snider K.H., Loeser J., Hesse A.M., Page C.E., Pelz C., Arthur J.S., Impey S. (2016). Targeted deletion of miR-132/-212 impairs memory and alters the hippocampal transcriptome. Learn. Mem..

[49] Levin E.D., McClernon F.J., Rezvani A.H. (2006). Nicotinic effects on cognitive function: Behavioral characterization, pharmacological specification, and anatomic localization. Psychopharmacology.

[50] Mineur Y.S., Fote G.M., Blakeman S., Cahuzac E.L., Newbold S.A., Picciotto M.R. (2016). Multiple Nicotinic Acetylcholine Receptor Subtypes in the Mouse Amygdala Regulate Affective Behaviors and Response to Social Stress. Neuropsychopharmacology.

[51] Dehkordi O., Rose J.E., Asadi S., Manaye K.F., Millis R.M., Jayam-Trouth A. (2015). Neuroanatomical circuitry mediating the sensory impact of nicotine in the central nervous system. J. Neurosci. Res..

[52] Hong L.E., Hodgkinson C.A., Yang Y., Sampath H., Ross T.J., Buchholz B., Salmeron B.J., Srivastava V., Thaker G.K., Goldman D. (2010). A genetically modulated, intrinsic cingulate circuit supports human nicotine addiction. Proc. Natl. Acad. Sci. USA.

[53] Zarrindast M.R., Khakpai F. (2019). The modulatory role of nicotine on cognitive and non-cognitive functions. Brain Res..

[54] Shaked I., Meerson A., Wolf Y., Avni R., Greenberg D., Gilboa-Geffen A., Soreq H. (2009). MicroRNA-132 potentiates cholinergic anti-inflammatory signaling by targeting acetylcholinesterase. Immunity.

[55] Lykhmus O., Mishra N., Koval L., Kalashnyk O., Gergalova G., Uspenska K., Komisarenko S., Soreq H., Skok M. (2016). Molecular Mechanisms Regulating LPS-Induced Inflammation in the Brain. Front. Mol. Neurosci..

[56] Mishra N., Friedson L., Hanin G., Bekenstein U., Volovich M., Bennett E.R., Greenberg D.S., Soreq H. (2017). Antisense miR-132 blockade via the AChE-R splice variant mitigates cortical inflammation. Sci. Rep..

[57] Wibrand K., Panja D., Tiron A., Ofte M.L., Skaftnesmo K.O., Lee C.S., Pena J.T., Tuschl T., Bramham C.R. (2010). Differential regulation of mature and precursor microRNA expression by NMDA and metabotropic glutamate receptor activation during LTP in the adult dentate gyrus in vivo. Eur. J. Neurosci..

[58] Hernandez-Rapp J., Rainone S., Goupil C., Dorval V., Smith P.Y., Saint-Pierre M., Vallee M., Planel E., Droit A., Calon F. (2016). microRNA-132/212 deficiency enhances Abeta production and senile plaque deposition in Alzheimer’s disease triple transgenic mice. Sci. Rep..

[59] Nagy V., Hollstein R., Pai T.P., Herde M.K., Buphamalai P., Moeseneder P., Lenartowicz E., Kavirayani A., Korenke G.C., Kozieradzki I. (2019). HACE1 deficiency leads to structural and functional neurodevelopmental defects. Neurol. Genet..

[60] Monje F.J., Kim E.J., Pollak D.D., Cabatic M., Li L., Baston A., Lubec G. (2012). Focal adhesion kinase regulates neuronal growth, synaptic plasticity and hippocampus-dependent spatial learning and memory. Neuro-Signals.

[61] Cicvaric A., Yang J., Bulat T., Zambon A., Dominguez-Rodriguez M., Kuhn R., Sadowicz M.G., Siwert A., Egea J., Pollak D.D. (2018). Enhanced synaptic plasticity and spatial memory in female but not male FLRT2-haplodeficient mice. Sci. Rep..

[62] Cicvaric A., Yang J., Krieger S., Khan D., Kim E.J., Dominguez-Rodriguez M., Cabatic M., Molz B., Acevedo Aguilar J.P., Milicevic R. (2016). The brain-tumor related protein podoplanin regulates synaptic plasticity and hippocampus-dependent learning and memory. Ann. Med..

[63] Halff A.W., Gomez-Varela D., John D., Berg D.K. (2014). A novel mechanism for nicotinic potentiation of glutamatergic synapses. J. Neurosci..

[64] Szabo S.I., Zelles T., Vizi E.S., Lendvai B. (2008). The effect of nicotine on spiking activity and Ca2+ dynamics of dendritic spines in rat CA1 pyramidal neurons. Hippocampus.

[65] Galvez B., Gross N., Sumikawa K. (2016). Activation of alpha7 nicotinic acetylcholine receptors protects potentiated synapses from depotentiation during theta pattern stimulation in the hippocampal CA1 region of rats. Neuropharmacology.

[66] Prestori F., Bonardi C., Mapelli L., Lombardo P., Goselink R., De Stefano M.E., Gandolfi D., Mapelli J., Bertrand D., Schonewille M. (2013). Gating of long-term potentiation by nicotinic acetylcholine receptors at the cerebellum input stage. PLoS ONE.

[67] Kim J.H., Udo H., Li H.L., Youn T.Y., Chen M., Kandel E.R., Bailey C.H. (2003). Presynaptic activation of silent synapses and growth of new synapses contribute to intermediate and long-term facilitation in Aplysia. Neuron.

[68] Bliss T.V., Lomo T. (1973). Long-lasting potentiation of synaptic transmission in the dentate area of the anaesthetized rabbit following stimulation of the perforant path. J. Physiol..

[69] Bear M.F., Abraham W.C. (1996). Long-term depression in hippocampus. Annu. Neurosci..

[70] Lomo T. (2003). The discovery of long-term potentiation. Philos. Trans. R. Soc. Lond..

[71] Andersen P., Lomo T. (1967). Control of hippocampal output by afferent volley frequency. Prog. Brain Res..

[72] Asaka Y., Jugloff D.G., Zhang L., Eubanks J.H., Fitzsimonds R.M. (2006). Hippocampal synaptic plasticity is impaired in the Mecp2-null mouse model of Rett syndrome. Neurobiol. Dis..

[73] Austin K.B., Bronzino J.D., Morgane P.J. (1989). Paired-pulse facilitation and inhibition in the dentate gyrus is dependent on behavioral state. Exp. Brain Res..

[74] Bekenstein J.W., Lothman E.W. (1991). Electrophysiological characterization of associational pathway terminating on dentate gyrus granule cells in the rat. Hippocampus.

[75] Kuhnt U., Voronin L.L. (1994). Interaction between paired-pulse facilitation and long-term potentiation in area CA1 of guinea-pig hippocampal slices: Application of quantal analysis. Neuroscience.

[76] Gruart A., Benito E., Delgado-Garcia J.M., Barco A. (2012). Enhanced cAMP response element-binding protein activity increases neuronal excitability, hippocampal long-term potentiation, and classical eyeblink conditioning in alert behaving mice. J. Neurosci..

[77] Connor S.A., Wang Y.T. (2016). A Place at the Table: LTD as a Mediator of Memory Genesis. Neuroscientist.

[78] Lynch M.A. (2004). Long-term potentiation and memory. Physiol. Rev..

[79] Lessmann V., Heumann R. (1998). Modulation of unitary glutamatergic synapses by neurotrophin-4/5 or brain-derived neurotrophic factor in hippocampal microcultures: Presynaptic enhancement depends on pre-established paired-pulse facilitation. Neuroscience.

[80] Capron B., Sindic C., Godaux E., Ris L. (2006). The characteristics of LTP induced in hippocampal slices are dependent on slice-recovery conditions. Learn. Mem..

[81] Nguyen P.V., Kandel E.R. (1997). Brief theta-burst stimulation induces a transcription-dependent late phase of LTP requiring cAMP in area CA1 of the mouse hippocampus. Learn. Mem..

[82] Hernandez R.V., Navarro M.M., Rodriguez W.A., Martinez J.L., LeBaron R.G. (2005). Differences in the magnitude of long-term potentiation produced by theta burst and high frequency stimulation protocols matched in stimulus number. Brain Res. Brain Res. Protoc..

[83] Malenka R.C., Bear M.F. (2004). LTP and LTD: An embarrassment of riches. Neuron.

[84] Sajikumar S., Frey J.U. (2004). Late-associativity, synaptic tagging, and the role of dopamine during LTP and LTD. Neurobiol. Learn. Mem..

[85] Sweatt J.D. (2003). Chapter 6—The Biochemistry of LTP Induction. Mechanisms of Memory.

[86] Gruart A., Leal-Campanario R., Lopez-Ramos J.C., Delgado-Garcia J.M. (2015). Functional basis of associative learning and its relationships with long-term potentiation evoked in the involved neural circuits: Lessons from studies in behaving mammals. Neurobiol. Learn. Mem..

[87] Gruart A., Munoz M.D., Delgado-Garcia J.M. (2006). Involvement of the CA3-CA1 synapse in the acquisition of associative learning in behaving mice. J. Neurosci..

[88] Gruart A., Sanchez-Campusano R., Fernandez-Guizan A., Delgado-Garcia J.M. (2015). A Differential and Timed Contribution of Identified Hippocampal Synapses to Associative Learning in Mice. Cereb. Cortex.

[89] Fukazawa Y., Saitoh Y., Ozawa F., Ohta Y., Mizuno K., Inokuchi K. (2003). Hippocampal LTP is accompanied by enhanced F-actin content within the dendritic spine that is essential for late LTP maintenance in vivo. Neuron.

[90] Stepan J., Dine J., Fenzl T., Polta S.A., von Wolff G., Wotjak C.T., Eder M. (2012). Entorhinal theta-frequency input to the dentate gyrus trisynaptically evokes hippocampal CA1 LTP. Front. Neural Circuits.

[91] Brown A.L., Flynn J.R., Smith D.W., Dayas C.V. (2011). Down-regulated striatal gene expression for synaptic plasticity-associated proteins in addiction and relapse vulnerable animals. Int. J. Neuropsychopharmacol..

[92] Christie M.J. (2008). Cellular neuroadaptations to chronic opioids: Tolerance, withdrawal and addiction. Br. J. Pharmacol..

[93] Jones S., Bonci A. (2005). Synaptic plasticity and drug addiction. Curr. Opin. Pharm..

[94] Kauer J.A., Malenka R.C. (2007). Synaptic plasticity and addiction. Nat. Rev..

[95] Nestler E.J. (2002). Common molecular and cellular substrates of addiction and memory. Neurobiol. Learn. Mem..

[96] Thomas M.J., Kalivas P.W., Shaham Y. (2008). Neuroplasticity in the mesolimbic dopamine system and cocaine addiction. Br. J. Pharmacol..

[97] Wolf M.E. (2002). Addiction: Making the connection between behavioral changes and neuronal plasticity in specific pathways. Mol. Interv..

[98] Kang E., Wen Z., Song H., Christian K.M., Ming G.L. (2016). Adult Neurogenesis and Psychiatric Disorders. Cold Spring Harb. Perspect. Biol..

[99] Abrous D.N., Adriani W., Montaron M.F., Aurousseau C., Rougon G., Le Moal M., Piazza P.V. (2002). Nicotine self-administration impairs hippocampal plasticity. J. Neurosci..

[100] Erickson M.A., Maramara L.A., Lisman J. (2010). A single brief burst induces GluR1-dependent associative short-term potentiation: A potential mechanism for short-term memory. J. Cogn. Neurosci..

[101] Larson J., Wong D., Lynch G. (1986). Patterned stimulation at the theta frequency is optimal for the induction of hippocampal long-term potentiation. Brain Res..

[102] Volianskis A., France G., Jensen M.S., Bortolotto Z.A., Jane D.E., Collingridge G.L. (2015). Long-term potentiation and the role of N-methyl-D-aspartate receptors. Brain Res..

[103] Chaarani B., Kan K.J., Mackey S., Spechler P.A., Potter A., Orr C., D’Alberto N., Hudson K.E., Banaschewski T., Bokde A.L.W. (2019). Low Smoking Exposure, the Adolescent Brain, and the Modulating Role of CHRNA5 Polymorphisms. Biol. Psychiatry Cogn. Neurosci. Neuroimaging.

[104] Treur J.L., Willemsen G., Bartels M., Geels L.M., van Beek J.H., Huppertz C., van Beijsterveldt C.E., Boomsma D.I., Vink J.M. (2015). Smoking During Adolescence as a Risk Factor for Attention Problems. Biol. Psychiatry.

[105] Smith R.F., McDonald C.G., Bergstrom H.C., Ehlinger D.G., Brielmaier J.M. (2015). Adolescent nicotine induces persisting changes in development of neural connectivity. Neurosci. Biobehav. Rev..

[106] Soderstrom K., Qin W., Williams H., Taylor D.A., McMillen B.A. (2007). Nicotine increases FosB expression within a subset of reward- and memory-related brain regions during both peri- and post-adolescence. Psychopharmacology.

[107] Trauth J.A., Seidler F.J., McCook E.C., Slotkin T.A. (1999). Adolescent nicotine exposure causes persistent upregulation of nicotinic cholinergic receptors in rat brain regions. Brain.

[108] Le Foll B., Goldberg S.R. (2009). Effects of nicotine in experimental animals and humans: An update on addictive properties. Handb. Exp. Pharmacol..

[109] Lynch W.J., Nicholson K.L., Dance M.E., Morgan R.W., Foley P.L. (2010). Animal models of substance abuse and addiction: Implications for science, animal welfare, and society. Comp. Med..

[110] O’Dell L.E., Khroyan T.V. (2009). Rodent models of nicotine reward: What do they tell us about tobacco abuse in humans?. Pharmacol. Biochem. Behav..

[111] Ijomone O.M., Nwoha P.U. (2015). Nicotine inhibits hippocampal and striatal acetylcholinesterase activities, and demonstrates dual action on adult neuronal proliferation and maturation. Pathophysiology.

[112] Fujii S., Jia Y., Yang A., Sumikawa K. (2000). Nicotine reverses GABAergic inhibition of long-term potentiation induction in the hippocampal CA1 region. Brain Res..

[113] Fujii S., Sumikawa K. (2001). Nicotine accelerates reversal of long-term potentiation and enhances long-term depression in the rat hippocampal CA1 region. Brain Res..

[114] Fujii S., Sumikawa K. (2001). Acute and chronic nicotine exposure reverse age-related declines in the induction of long-term potentiation in the rat hippocampus. Brain.

[115] Ji D., Lape R., Dani J.A. (2001). Timing and location of nicotinic activity enhances or depresses hippocampal synaptic plasticity. Neuron.

[116] Mann E.O., Greenfield S.A. (2003). Novel modulatory mechanisms revealed by the sustained application of nicotine in the guinea-pig hippocampus in vitro. J. Physiol..

[117] Nakauchi S., Brennan R.J., Boulter J., Sumikawa K. (2007). Nicotine gates long-term potentiation in the hippocampal CA1 region via the activation of alpha2* nicotinic ACh receptors. Eur. J. Neurosci..

[118] Nakauchi S., Sumikawa K. (2012). Endogenously released ACh and exogenous nicotine differentially facilitate long-term potentiation induction in the hippocampal CA1 region of mice. Eur. J. Neurosci..

[119] Sawada S., Ohno-Shosaku T., Yamamoto C. (1994). Augmenting action of nicotine on population spikes in the dentate gyrus of the guinea pig. Neurosci. Res..

[120] Sawada S., Yamamoto C., Ohno-Shosaku T. (1994). Long-term potentiation and depression in the dentate gyrus, and effects of nicotine. Neurosci. Res..

[121] Gray R., Rajan A.S., Radcliffe K.A., Yakehiro M., Dani J.A. (1996). Hippocampal synaptic transmission enhanced by low concentrations of nicotine. Nature.

[122] Matsuyama S., Matsumoto A., Enomoto T., Nishizaki T. (2000). Activation of nicotinic acetylcholine receptors induces long-term potentiation in vivo in the intact mouse dentate gyrus. Eur. J. Neurosci..

[123] Fujii S., Ji Z., Morita N., Sumikawa K. (1999). Acute and chronic nicotine exposure differentially facilitate the induction of LTP. Brain Res..

[124] Alzoubi K.H., Srivareerat M., Tran T.T., Alkadhi K.A. (2013). Role of alpha7- and alpha4beta2-nAChRs in the neuroprotective effect of nicotine in stress-induced impairment of hippocampus-dependent memory. Int. J. Neuropsychopharmacol..

[125] Cheng Q., Yakel J.L. (2015). The effect of alpha7 nicotinic receptor activation on glutamatergic transmission in the hippocampus. Biochem. Pharm..

[126] Kenney J.W., Raybuck J.D., Gould T.J. (2012). Nicotinic receptors in the dorsal and ventral hippocampus differentially modulate contextual fear conditioning. Hippocampus.

[127] Lu C.B., Henderson Z. (2010). Nicotine induction of theta frequency oscillations in rodent hippocampus in vitro. Neuroscience.

[128] Melichercik A.M., Elliott K.S., Bianchi C., Ernst S.M., Winters B.D. (2012). Nicotinic receptor activation in perirhinal cortex and hippocampus enhances object memory in rats. Neuropharmacology.

[129] Parameshwaran K., Buabeid M.A., Karuppagounder S.S., Uthayathas S., Thiruchelvam K., Shonesy B., Dityatev A., Escobar M.C., Dhanasekaran M., Suppiramaniam V. (2012). Developmental nicotine exposure induced alterations in behavior and glutamate receptor function in hippocampus. Cell Life Sci..

[130] Birthelmer A., Stemmelin J., Jackisch R., Cassel J.C. (2003). Presynaptic modulation of acetylcholine, noradrenaline, and serotonin release in the hippocampus of aged rats with various levels of memory impairments. Brain Res. Bull..

[131] Sharp B.M., Yatsula M., Fu Y. (2004). Effects of galantamine, a nicotinic allosteric potentiating ligand, on nicotine-induced catecholamine release in hippocampus and nucleus accumbens of rats. J. Pharm. Exp..

[132] Abdel-Rahman A., Dechkovskaia A., Mehta-Simmons H., Guan X., Khan W., Abou-Donia M. (2003). Increased expression of glial fibrillary acidic protein in cerebellum and hippocampus: Differential effects on neonatal brain regional acetylcholinesterase following maternal exposure to combined chlorpyrifos and nicotine. J. Toxicol. Environ. Health Part A.

[133] Jamal M., Ameno K., Miki T., Tanaka N., Ohkubo E., Kinoshita H. (2010). Effects of systemic nicotine, alcohol or their combination on cholinergic markers in the frontal cortex and hippocampus of rat. Neurochem. Res..

[134] Perry E.K., Court J.A., Johnson M., Smith C.J., James V., Cheng A.V., Kerwin J.M., Morris C.M., Piggott M.A., Edwardson J.A. (1993). Autoradiographic comparison of cholinergic and other transmitter receptors in the normal human hippocampus. Hippocampus.

[135] Simchovitz A., Heneka M.T., Soreq H. (2017). Personalized genetics of the cholinergic blockade of neuroinflammation. J. Neurochem..

[136] Alarcon J.M., Malleret G., Touzani K., Vronskaya S., Ishii S., Kandel E.R., Barco A. (2004). Chromatin acetylation, memory, and LTP are impaired in CBP+/− mice: A model for the cognitive deficit in Rubinstein-Taybi syndrome and its amelioration. Neuron.

[137] Kang H., Sun L.D., Atkins C.M., Soderling T.R., Wilson M.A., Tonegawa S. (2001). An important role of neural activity-dependent CaMKIV signaling in the consolidation of long-term memory. Cell.

[138] Alkadhi K.A., Alzoubi K.H., Srivareerat M., Tran T.T. (2011). Chronic psychosocial stress exacerbates impairment of synaptic plasticity in beta-amyloid rat model of Alzheimer’s disease: Prevention by nicotine. Curr. Alzheimer Res..

[139] Haghparast A., Taslimi Z., Ramin M., Azizi P., Khodagholi F., Hassanpour-Ezatti M. (2011). Changes in phosphorylation of CREB, ERK, and c-fos induction in rat ventral tegmental area, hippocampus and prefrontal cortex after conditioned place preference induced by chemical stimulation of lateral hypothalamus. Behav. Brain Res..

[140] Kenney J.W., Poole R.L., Adoff M.D., Logue S.F., Gould T.J. (2012). Learning and nicotine interact to increase CREB phosphorylation at the jnk1 promoter in the hippocampus. PLoS ONE.

[141] Magill S.T., Cambronne X.A., Luikart B.W., Lioy D.T., Leighton B.H., Westbrook G.L., Mandel G., Goodman R.H. (2010). microRNA-132 regulates dendritic growth and arborization of newborn neurons in the adult hippocampus. Proc. Natl. Acad. Sci. USA.

[142] Hansen K.F., Sakamoto K., Wayman G.A., Impey S., Obrietan K. (2010). Transgenic miR132 alters neuronal spine density and impairs novel object recognition memory. PLoS ONE.

[143] Vo N., Klein M.E., Varlamova O., Keller D.M., Yamamoto T., Goodman R.H., Impey S. (2005). A cAMP-response element binding protein-induced microRNA regulates neuronal morphogenesis. Proc. Natl. Acad. Sci. USA.

[144] Cao G., Zhu J., Zhong Q., Shi C., Dang Y., Han W., Liu X., Xu M., Chen T. (2013). Distinct roles of methamphetamine in modulating spatial memory consolidation, retrieval, reconsolidation and the accompanying changes of ERK and CREB activation in hippocampus and prefrontal cortex. Neuropharmacology.

[145] Ko Y.H., Kwon S.H., Lee S.Y., Jang C.G. (2017). Liquiritigenin ameliorates memory and cognitive impairment through cholinergic and BDNF pathways in the mouse hippocampus. Arch. Pharm. Res..

[146] Liu L., Zhu J., Zhou L., Wan L. (2016). RACK1 promotes maintenance of morphine-associated memory via activation of an ERK-CREB dependent pathway in hippocampus. Sci. Rep..

[147] Leal G., Comprido D., Duarte C.B. (2014). BDNF-induced local protein synthesis and synaptic plasticity. Neuropharmacology.

[148] Zagaar M., Dao A., Levine A., Alhaider I., Alkadhi K. (2013). Regular exercise prevents sleep deprivation associated impairment of long-term memory and synaptic plasticity in the CA1 area of the hippocampus. Sleep.

[149] Lambert T.J., Storm D.R., Sullivan J.M. (2010). MicroRNA132 modulates short-term synaptic plasticity but not basal release probability in hippocampal neurons. PLoS ONE.

[150] Scott H.L., Tamagnini F., Narduzzo K.E., Howarth J.L., Lee Y.B., Wong L.F., Brown M.W., Warburton E.C., Bashir Z.I., Uney J.B. (2012). MicroRNA-132 regulates recognition memory and synaptic plasticity in the perirhinal cortex. Eur. J. Neurosci..

[151] Balazsfi D., Farkas L., Csikota P., Fodor A., Zsebok S., Haller J., Zelena D. (2016). Sex-dependent role of vesicular glutamate transporter 3 in stress-regulation and related anxiety phenotype during the early postnatal period. Stress.

[152] Caruso M.J., Crowley N.A., Reiss D.E., Caulfield J.I., Luscher B., Cavigelli S.A., Kamens H.M. (2018). Adolescent Social Stress Increases Anxiety-like Behavior and Alters Synaptic Transmission, Without Influencing Nicotine Responses, in a Sex-Dependent Manner. Neuroscience.

[153] Chmielarz P., Kreiner G., Nalepa I. (2015). Selective ablation of glucocorticoid receptors in the noradrenergic system affects evening corticosterone levels in a sex-dependent manner. Pharm. Rep..

[154] Grech A.M., Ratnayake U., Hannan A.J., van den Buuse M., Hill R.A. (2018). Sex-Dependent Effects of Environmental Enrichment on Spatial Memory and Brain-Derived Neurotrophic Factor (BDNF) Signaling in a Developmental “Two-Hit” Mouse Model Combining BDNF Haploinsufficiency and Chronic Glucocorticoid Stimulation. Front. Behav. Neurosci..

[155] Becker J.B., Chartoff E. (2019). Sex differences in neural mechanisms mediating reward and addiction. Neuropsychopharmacology.

[156] Gillies G.E., Pienaar I.S., Vohra S., Qamhawi Z. (2014). Sex differences in Parkinson’s disease. Front. Neuroendocr..

[157] Gillies G.E., Virdee K., McArthur S., Dalley J.W. (2014). Sex-dependent diversity in ventral tegmental dopaminergic neurons and developmental programing: A molecular, cellular and behavioral analysis. Neuroscience.

[158] Williams O.O.F., Coppolino M., George S.R., Perreault M.L. (2021). Sex Differences in Dopamine Receptors and Relevance to Neuropsychiatric Disorders. Brain Sci..

[159] Pollak D.D., John J., Bubna-Littitz H., Schneider A., Hoeger H., Lubec G. (2006). Components of the protein quality control system are expressed in a strain-dependent manner in the mouse hippocampus. Neurochem. Int..

[160] Pollak D.D., John J., Scharl T., Leisch F., Schneider A., Hoeger H., Lubec G. (2006). Strain-dependent regulation of neurotransmission and actin-remodelling proteins in the mouse hippocampus. Genes Brain Behav..

[161] Pollak D.D., John J., Schneider A., Hoeger H., Lubec G. (2006). Strain-dependent expression of signaling proteins in the mouse hippocampus. Neuroscience.

[162] Pollak D.D., Scharl T., Leisch F., Herkner K., Villar S.R., Hoeger H., Lubec G. (2005). Strain-dependent regulation of plasticity-related proteins in the mouse hippocampus. Behav. Brain Res..

